# Association between red blood cell distribution width-to-albumin ratio and cardiovascular disease mortality and all-cause mortality in patients with hypertension: An observational study

**DOI:** 10.1097/MD.0000000000044676

**Published:** 2025-09-26

**Authors:** Mengmeng Shan, Suling Ye, Yingying Chen, Shaomi Li, Jinzhou Zhu, Jie Li

**Affiliations:** a Department of Cardiology, Ruijin-Hainan Hospital Shanghai Jiao Tong University School of Medicine (Hainan Boao Research Hospital), Qionghai, China.

**Keywords:** hypertension, mortality, NHANES, red blood cell distribution width-to-albumin ratio, regression analysis

## Abstract

This study aimed to investigate the relationship between red blood cell distribution width to-albumin ratio (RAR) and mortality risk among patients with hypertension, thereby providing a foundation for early assessment in this population. A total of 6830 adult patients with hypertension were included in this study. To explore the association between RAR levels and 10-year all-cause mortality in patients with hypertension, smooth curve fitting and Kaplan–Meier (KM) curve analysis were employed. Additionally, Cox regression analysis was conducted to evaluate the impact of RAR on all-cause mortality. The robustness of the findings was confirmed through stratified analysis and interaction tests. A competing risk model was also utilized to assess the relationship between RAR and cardiovascular as well as cerebrovascular mortality in patients with hypertension. The result of smooth curve fitting showed that 10-year all-cause mortality rate among patients with hypertension progressively increased with higher RAR levels. After fully adjusting for confounding factors, RAR remained significantly associated with a 10-year all-cause mortality risk in patients with hypertension (HR = 1.69, 95% confidence interval: 1.50–1.8, *P* < .001). Furthermore, the competing risk model revealed a similar association between RAR and both cardiovascular and cerebrovascular mortality in this group. With RAR levels rise, the risk of all-cause mortality and mortality due to cardiovascular disease also escalates. This study suggest that RAR may serve as a useful tool for assessing the prognosis of patients with hypertension.

## 1. Introduction

According to the World Health Organization (2023), the number of people with hypertension worldwide doubled from 650 million in 1990 to 1.3 billion in 2019, which affects over 30% of adults globally, underscoring the urgent need for enhanced control efforts.^[1]^ Hypertension is also a major risk factor for cardiovascular disease, as its onset and progression can lead to vascular endothelial damage. This damage, combined with the effects of renin-angiotensin-aldosterone system activation, inflammation, and immune factors, promotes the development of cardiovascular and cerebrovascular diseases. Statistics show that approximately 8.5 million people die each year from hypertension and its complications, particularly cardiovascular and cerebrovascular diseases, which places tremendous pressure on global health.^[1–3]^ Therefore, early identification of risk factors associated with death from hypertension is particularly important.

As one of the factors contributing to the pathogenesis of hypertension, the inflammatory response is involved in the regulation of hypertension as well as the initiation and progression of hypertension-related target organ damage.^[4–6]^ Immune dysregulation drives inflammatory processes that mediate vascular inflammation and microvascular remodeling, thereby promoting hypertension progression.^[6]^ Activated immune cells release proinflammatory factors – including interleukin-17, interferon-γ, tumor necrosis factor-α, and interleukin-6 – which exacerbate vascular pathological alterations through induction of oxidative stress and endothelial dysfunction.^[7,8]^ Although C-reactive protein (CRP) serves as a classical inflammatory biomarker associated with cardiovascular disease risk,^[9,10]^ its specificity is confounded by demographic and lifestyle variables.^[11]^ Furthermore, the requisite additional expenditure for CRP testing restricts its clinical utility in large-scale hypertension screening initiatives. The red blood cell distribution width (RDW)-to-albumin ratio (RAR) is a new biomarker of inflammation. Previous studies have shown that RAR is associated with the occurrence of various diseases and has predictive value for the risk of death from these diseases, especially cardiovascular and cerebrovascular diseases.^[12–16]^ Studies have found that both RDW and albumin are associated with the risk of developing hypertension.^[17–19]^ However, no studies have been conducted on the correlation between RAR and the prognosis of patients with hypertension. Therefore, this study aims to explore the relationship between RAR and the risk of death in patients with hypertension using the National Health and Nutrition Examination Survey (NHANES) database to provide a basis for the early assessment of patients with hypertension.

## 2. Methods

### 2.1. General information

The data for the study was obtained from the NHANES, a public database that monitors human health conditions and nutrition across ethnic groups in the United States through continuous updates.^[20]^ NHANES obtained resident samples of all races in the United States through multi-stage stratified sampling, including people of all ages from all regions of the country. Therefore, NHANES data is a representative reflection of the health status of United States residents. All data are available on the official website (https://wwwn.cdc.gov/nchs/nhanes/Default.aspx).

**Figure 1.**
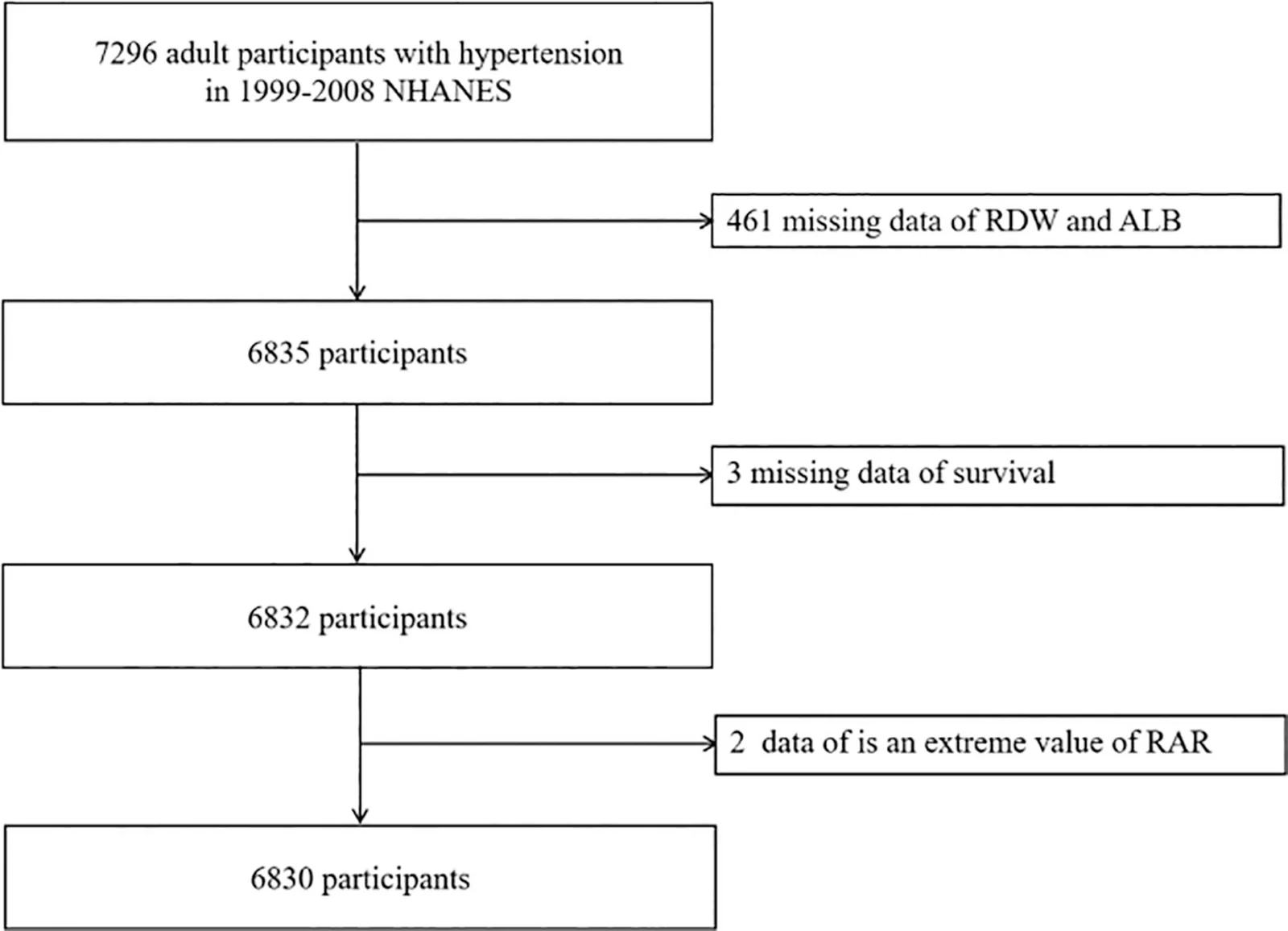
Flowchart of the inclusion and exclusion process for the study population.

From 1999 to 2008, a total of 7296 adult participants with hypertension from different ethnic groups and multiple regions were included in the NHANES database. Of these, 461 participants had missing information on RDW and albumin and were excluded. Three additional participants were missing follow-up results for vital status and were also excluded. In addition, 2 extreme values of RAR were excluded to ensure the reliability of the results. Consequently, a total of 5024 participants were included in this study (Fig. 1). Data perturbation techniques were utilized to reduce the risk of participant re-identification and maintain patient confidentiality.

### 2.2. Ethical statement

The study protocol received approval from the Ethics Review Committee of the National Center for Health Statistics in the United States, and all participant provided informed consent. All experiments involved in this paper are performed in accordance with relevant guidelines and regulations.

### 2.3. Definition and measurement of hypertension

The participants’ blood pressure was measured 3 times using a mercury sphygmomanometer, with an interval of >30 seconds between each measurement. The systolic blood pressure (SBP) and diastolic blood pressure (DBP) values were recorded separately, and the final blood pressure level was expressed as the mean value. Participants who did not have their blood pressure measured 3 times were excluded from this study. A questionnaire was administered to determine whether participants were taking antihypertensive medication. According to the European Cardiology Guidelines, a participant was defined as having hypertension if their blood pressure was ≥140/90 mm Hg^[21]^ or if they were taking antihypertensive medication. In sensitivity analyses, a patient was redefined as having hypertension if their blood pressure was ≥130/80 mm Hg^[22]^ or if the they were taking antihypertensive medication, based on the 2017 American Heart Association recommendations.

### 2.4. Survival status and follow-up time

Using the national death index, the National Center for Health Statistics recorded the survival status of participants with the aim of investigating the relationship between various health factors and mortality. Data were obtained from the official website (https://www.cdc.gov/nchs/nhanes/index.htm).

### 2.5. Collection of patients’ baseline data

The baseline data of the patients were obtained through a computer-assisted personal interview system, questionnaire survey, and blood tests. The data included gender, age, race, education level, family income to poverty ratio, body mass index (BMI), history of smoking, alcohol consumption, hypercholesterolemia, diabetes, heart failure, coronary heart disease, stroke, alanine aminotransferase (ALT), aspartate aminotransferase (AST), creatinine, uric acid, hemoglobin, iron, CRP, white blood cell (WBC), neutrophil, lymphocyte**,** RDW, and albumin. The presence of hypertension, hypercholesterolemia, diabetes, heart failure, and stroke were defined as having been diagnosed with these conditions by a doctor. Patients were defined as having a history of coronary heart disease if they answered “yes” to having been told by a physician that they had coronary heart disease, angina pectoris, or myocardial infarction. The RAR was calculated by dividing the RDW by albumin and expressed as both a continuous variable and an interquartile variable.

### 2.6. Statistical analysis

The differences in continuous variables between the survivor and death groups were compared using t-tests, while categorical variables were compared using chi-square tests. Smooth curve fitting and Kaplan–Meier (KM) curve analysis were utilized to explore the association trend between RAR level and 10-year all-cause mortality in patients with hypertension. The effect of RAR on all-cause mortality was assessed via Cox regression analysis. Receiver operating characteristic (ROC) curve is used to compare the predictive power of different inflammatory factors. Before conducting Cox regression, all covariates were screened for collinearity, with results indicating that the variance inflation factor for all covariates did not exceed 5. To adjust the confounders stepwise, 3 models were developed. No variables were adjusted in Model 1. Model 2 was adjusted for demographic data (including gender, age, and race). In Model 3, in addition to demographic information, the covariates that changed estimates of the effect of RAR on mortality by more than 10% were also adjusted (including age, gender, race, education level, the ratio of household income to poverty, BMI, smoking, alcohol, hypercholesterolemia, diabetes, heart failure, coronary heart disease, stroke, ALT, creatinine, uric acid, hemoglobin, iron, SBP and DBP). To investigate whether the results were modified by various comorbidities, subgroup analyses and interactive tests were conducted. Additionally, a competing risk model was employed to examine the effect of RAR on cardiovascular and cerebrovascular mortality in patients with hypertension. To ensure the reliability of the results, RAR was included in the regression calculations as both continuous and interquartile variables. In sensitivity analyses, hypertension was redefined using a blood pressure criterion of 130/80 mm Hg, and new participants were screened.

This study used EmpowerXYS to analyze the data, with a *P*-value <.05 indicating statistical significance.

## 3. Results

### 3.1. Baseline characteristics

A total of 6830 adult patients with hypertension were included in this study. After a mean follow-up duration of 149 months, patients were categorized into the survivor (n = 5087) and death (n = 1743) groups. Compared with the survivor group, patients in the death group were older, had lower ALT, hemoglobin, iron and albumin levels, higher creatinine, uric acid, and RDW levels, and were more likely to be male, smokers, and have diabetes, heart failure, coronary heart disease, and stroke. There were also differences in race, education level, household income to poverty ratio, and BMI. Results are presented in Table 1.

**Table 1:** Basic characteristics of participants.

Number	All	Survival	All-cause mortality	*P*
	6830	5087	1743	
Time, mo	149.0 ± 60.0	177.8 ± 35.2	65.2 ± 32.3	<.001
SBP	141.8 ± 21.2	140.2 ± 19.9	146.6 ± 23.9	<.001
DBP	73.8 ± 16.4	75.6 ± 15.4	68.3 ± 17.8	<.001
Age, yr	61.9 ± 14.2	58.6 ± 13.9	71.6 ± 10.1	<.001
Sex				
Male	3405 (49.9)	2438 (47.9)	967 (55.5)	<.001
Female	3425 (50.1)	2649 (52.1)	776 (44.5)	
Race				
Mexican American	1125 (16.5)	905 (17.8)	220 (12.6)	<.001
Other Hispanic	333 (4.9)	275 (5.4)	58 (3.3)	
Non-Hispanic White	3574 (52.3)	2486 (48.9)	1088 (62.4)	
Non-Hispanic Black	1590 (23.3)	1241 (24.4)	349 (20.0)	
Other Race	208 (3.0)	180 (3.5)	28 (1.6)	
Education level				
<High school	2430 (35.6)	1670 (32.8)	760 (43.6)	<.001
High school graduate or general equivalency diploma	1715 (25.1)	1282 (25.2)	433 (24.8)	
>High school	2674 (39.2)	2132 (41.9)	542 (31.1)	
Unknown	11 (0.2)	3 (0.1)	8 (0.5)	
Ratio of family income to poverty				
≤1	1074 (15.7)	751 (14.8)	323 (18.5)	<.001
1–3	2919 (42.7)	2006 (39.4)	913 (52.4)	
>3	2296 (33.6)	1941 (38.2)	355 (20.4)	
Unknown	541 (7.9)	389 (7.6)	152 (8.7)	
BMI				
<30 kg/m^2^	3138 (45.9)	2215 (43.5)	923 (53.0)	<.001
≧30 kg/m^2^	2323 (34.0)	1890 (37.2)	433 (24.8)	
Unknown	1369 (20.0)	982 (19.3)	387 (22.2)	
Smoking				
No	3380 (49.5)	2675 (52.6)	705 (40.4)	<.001
Yes	3450 (50.5)	2412 (47.4)	1038 (59.6)	
Alcohol use				
No	2605 (38.1)	1906 (37.5)	699 (40.1)	.051
Yes	4225 (61.9)	3181 (62.5)	1044 (59.9)	
Hypercholesterolemia				
No	3822 (56.0)	2833 (55.7)	989 (56.7)	.446
Yes	3008 (44.0)	2254 (44.3)	754 (43.3)	
Diabetes				
No	5463 (80.0)	4207 (82.7)	1256 (72.1)	<.001
Yes	1367 (20.0)	880 (17.3)	487 (27.9)	
Heart failure				
No	6407 (93.8)	4888 (96.1)	1519 (87.1)	<.001
Yes	423 (6.2)	199 (3.9)	224 (12.9)	
Cardiovascular disease				
No	5793 (84.8)	4517 (88.8)	1276 (73.2)	<.001
Yes	1037 (15.2)	570 (11.2)	467 (26.8)	
Stroke				
No	6345 (92.9)	4854 (95.4)	1491 (85.5)	<.001
Yes	485 (7.1)	233 (4.6)	252 (14.5)	
ALT, U/L	25.2 ± 16.2	26.3 ± 16.3	22.0 ± 15.5	<.001
AST, U/L	25.9 ± 12.8	25.9 ± 11.8	26.1 ± 15.3	.008
Creatinine, µmol/L	87.1 ± 57.9	81.4 ± 45.0	103.7 ± 82.7	<.001
Uric acid, µmol/L	347.3 ± 90.4	342.9 ± 85.5	360.1 ± 102.1	<.001
Hemoglobin, g/dL	14.2 ± 1.5	14.3 ± 1.5	13.9 ± 1.6	<.001
Iron, µmol/L	15.0 ± 5.9	15.2 ± 5.8	14.5 ± 6.2	<.001
CRP, mg/Dl	0.5 ± 0.9	0.5 ± 0.8	0.6 ± 1.2	<.001
WBC, 10^3^/µL	7.3 ± 2.5	7.2 ± 2.1	7.5 ± 3.4	<.001
Neutrophil, 10^3^/µL	4.3 ± 1.6	4.2 ± 1.6	4.6 ± 1.8	<.001
Lymphocyte, 10^3^/µL	2.1 ± 1.6	2.2 ± 1.0	2.0 ± 2.6	.004
RDW, %	13.0 ± 1.2	12.9 ± 1.0	13.5 ± 1.5	<.001
Albumin, g/dL	4.2 ± 0.3	4.2 ± 0.3	4.1 ± 0.3	<.001
RAR continuous	3.1 ± 0.4	3.1 ± 0.4	3.3 ± 0.5	<.001
RAR quartile				
Q1 (2.09–2.80)	1489 (21.8)	1257 (24.7)	232 (13.3)	<.001
Q2 (2.80–3.03)	1866 (27.3)	1523 (29.9)	343 (19.7)	
Q3 (3.03–3.26)	1558 (22.8)	1132 (22.3)	426 (24.4)	
Q4 (3.26–7.56)	1917 (28.1)	1175 (23.1)	742 (42.6)	

ALT = alanine aminotransferase, AST = aspartate aminotransferase, BMI = body mass index, CRP = C-reactive protein, DBP = diastolic blood pressure, RAR = red blood cell distribution width-to-albumin ratio, RDW = red blood cell distribution width, SBP = systolic blood pressure, WBC = white blood cell.

### 3.2. Correlation analysis between RAR and 10-year all-cause mortality in patients with hypertension

**Figure 2.**
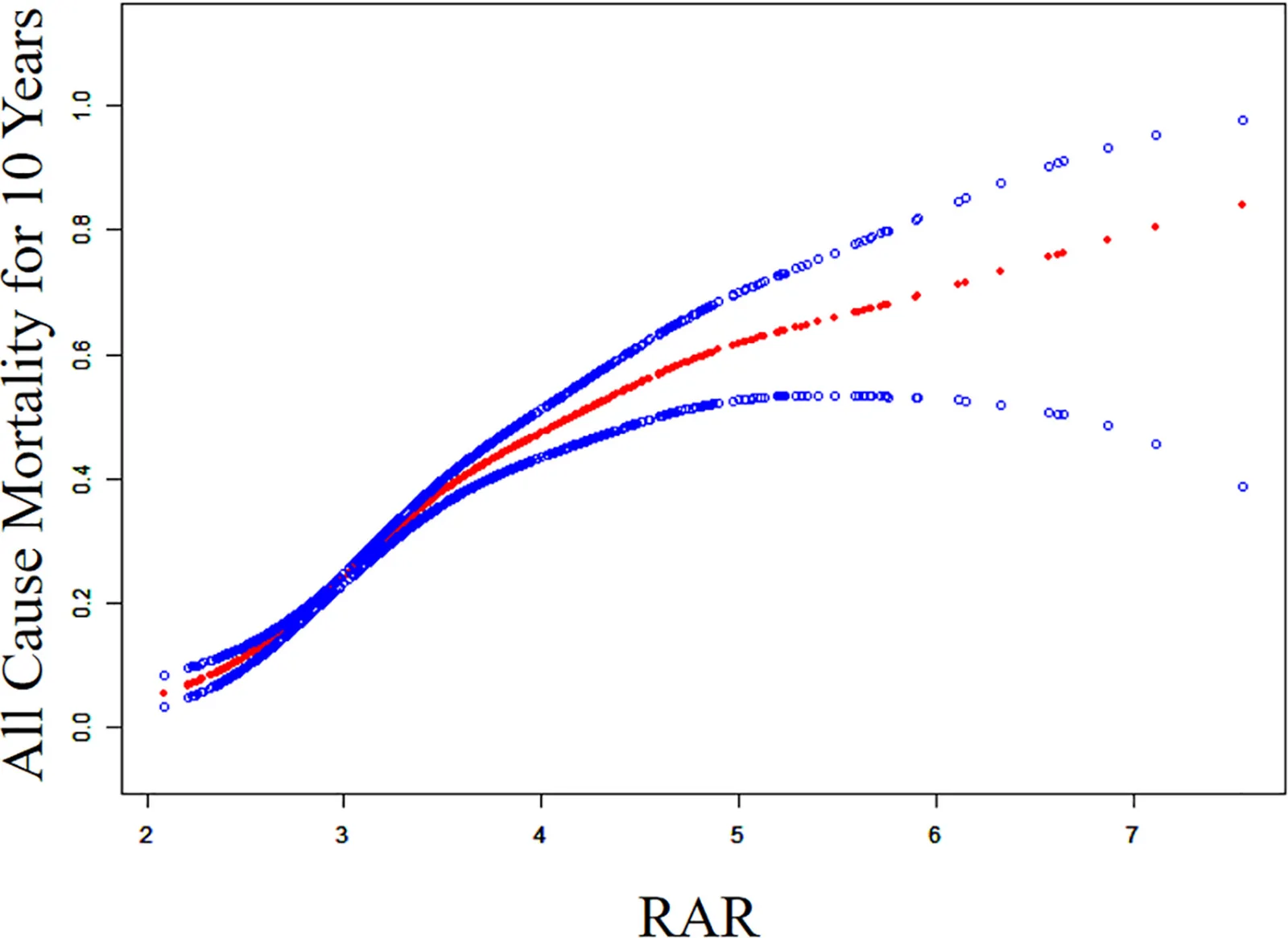
Smoothing curve fitting between RAR and all-cause mortality for 10 yr. RAR = red blood cell distribution width-to-albumin ratio.

**Figure 3.**
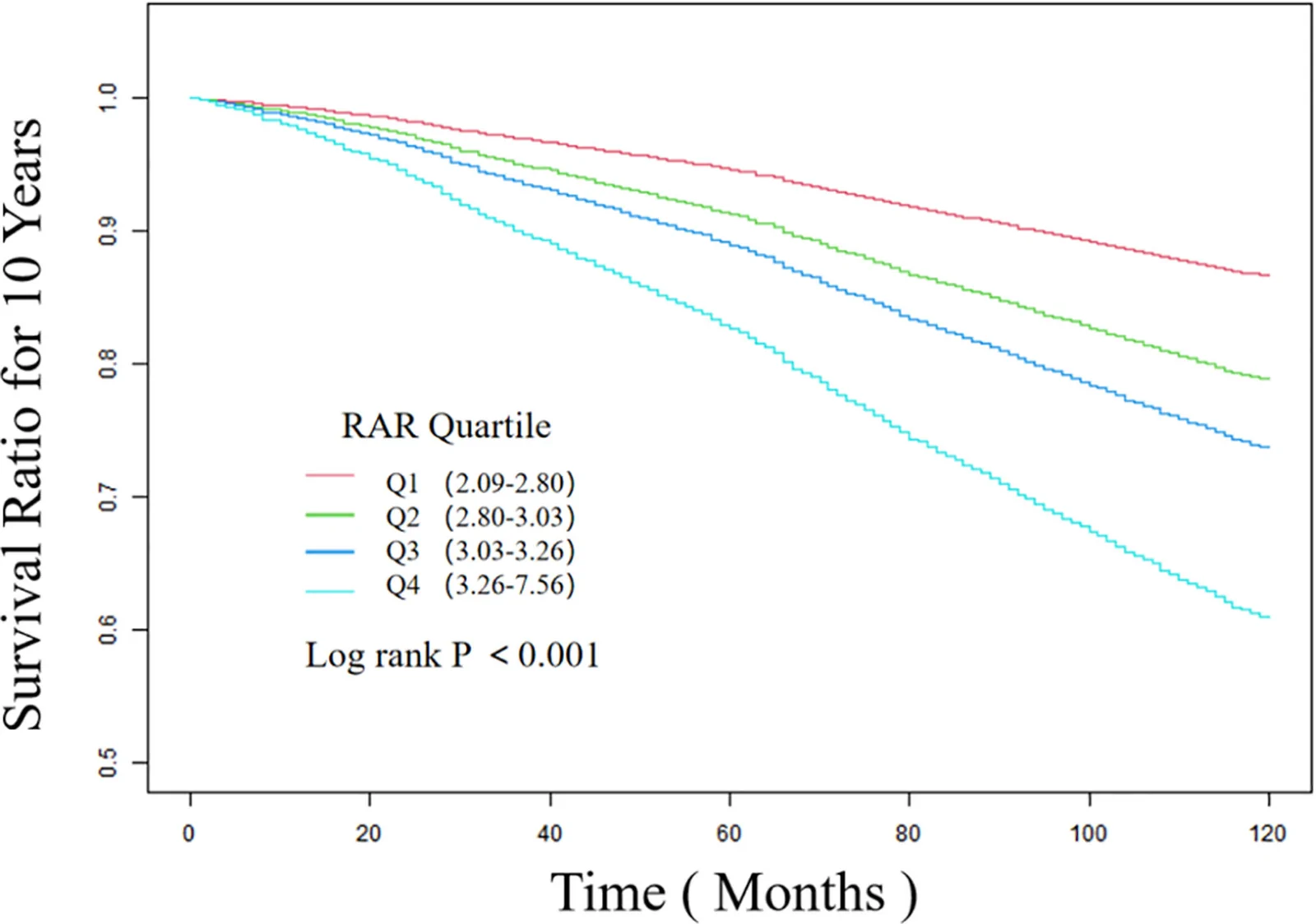
KM curves between RAR levels and survival ratio for 10 yr. KM = Kaplan–Meier, RAR = red blood cell distribution width-to-albumin ratio.

**Figure 4.**
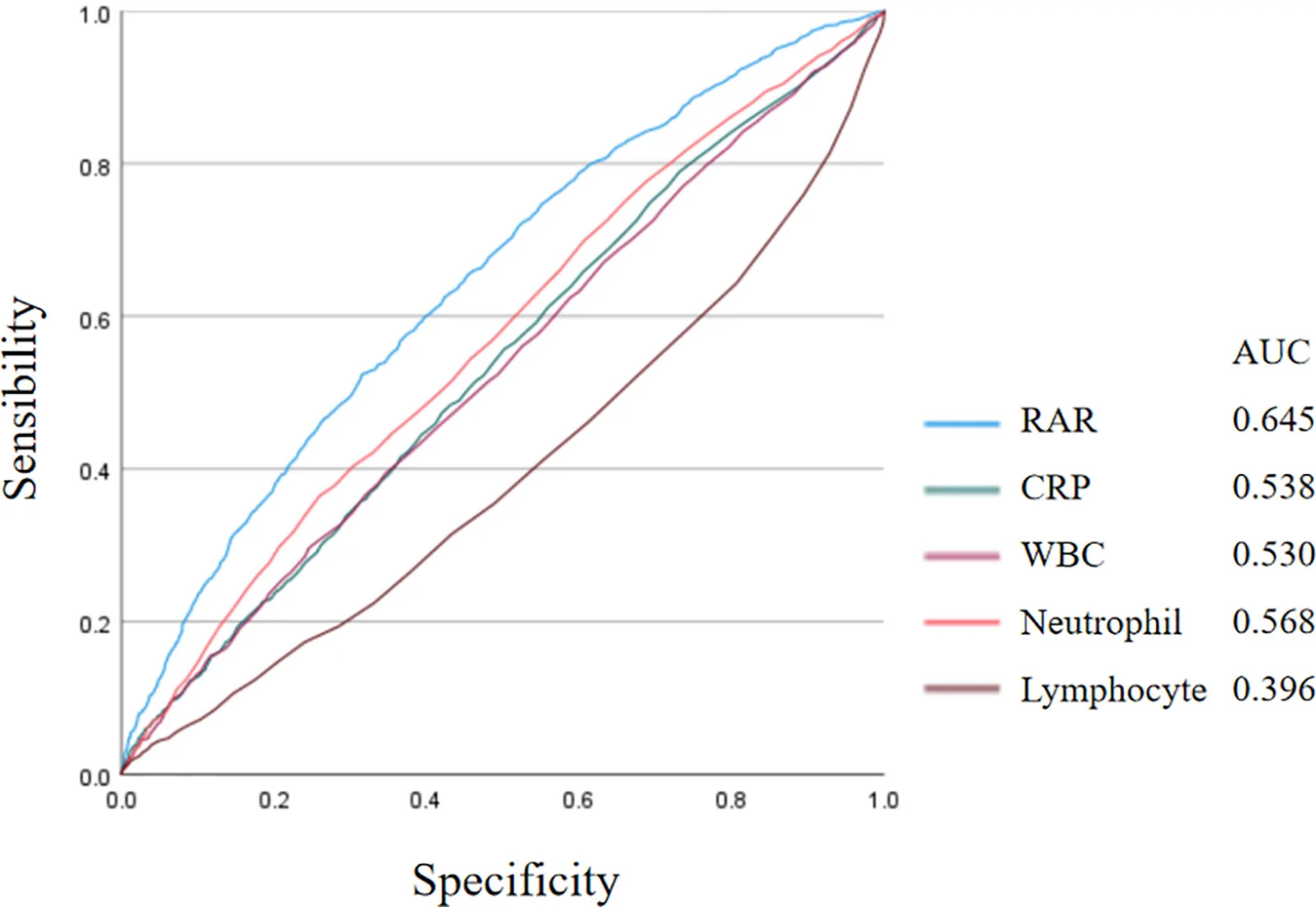
ROC curve of different inflammatory factors. AUC = area under curve, ROC = receiver operating characteristic.

The smooth curve indicated that as RAR levels increased, the 10-year all-cause mortality rate among patients with hypertension also gradually increased (Fig. 2). The KM curve further suggested that higher RAR levels were associated with lower survival rates in these patients (Fig. 3). Table 2 presents the results of the Cox regression analysis examining the relationship between RAR and 10-year all-cause mortality. After fully adjusting for confounding factors, including age, gender, race, education level, the ratio of household income to poverty, BMI, smoking, alcohol, hypercholesterolemia, diabetes, heart failure, coronary heart disease, stroke, ALT, creatinine, uric acid, hemoglobin, iron, SBP and DBP, RAR remained significantly associated with 10-year all-cause mortality (hazard ratio [HR] = 1.69; 95% confidence interval [CI] = 1.50–1.89; *P* < .001). The interquartile variable analysis also indicated that higher RAR levels increased the risk of death in patients with hypertension (HR = 2.05; 95% CI: 1.68–2.49; *P* < .001, *P* for trend <.001). Compared with other traditional inflammatory factors such as CRP, WBC, neutrophil and lymphocyte, RAR showed a more significant correlation. ROC curve showed that RAR has better prediction value (Fig. 4).

**Table 2:** Cox regression analysis between RAR and all-cause mortality.

	Model 1	Model 2	Model 3
	HR (95% CI) *P*	HR (95% CI) *P*	HR (95% CI) *P*
CRP	1.14 (1.10, 1.18) < .001	1.17 (1.14, 1.21) <.001	1.10 (1.05, 1.16) <.001
WBC	1.03 (1.02, 1.04) <.001	1.03 (1.02, 1.04) <.001	1.02 (1.01, 1.04) .003
Neutrophil	1.13 (1.10, 1.16) <.001	1.19 (1.16, 1.22) <.001	1.12 (1.08, 1.16) <.001
Lymphocyte	0.84 (0.79, 0.90) <.001	1.00 (0.97, 1.03) .915	0.97 (0.93, 1.02) .265
RDW	1.28 (1.25, 1.31) <.001	1.26 (1.23, 1.30) <.001	1.16 (1.12, 1.21) <.001
Albumin	0.42 (0.37, 0.49) <.001	0.41 (0.35, 0.48) <.001	0.58 (0.48, 0.70) <.001
RAR continuous	2.12 (1.98, 2.28) <.001	2.13 (1.98, 2.30) <.001	1.69 (1.50, 1.89) <0.001
RAR quartile			
Q1 (2.09–2.80)	Ref.	Ref.	Ref.
Q2 (2.80–3.03)	1.60 (1.35, 1.89) <.001	1.28 (1.08, 1.51) .005	1.18 (0.97, 1.43) .095
Q3 (3.03–3.26)	2.08 (1.75, 2.45) <.001	1.62 (1.37, 1.92) <.001	1.32 (1.08, 1.61) .006
Q4 (3.26–7.56)	3.35 (2.87, 3.91) <.001	2.68 (2.28, 3.14) <.001	2.05 (1.68, 2.49) <.001
*P* trend	<.001	<.001	<.001

Model 1: Unadjusted for any covariates; Model 2: adjusted for age, gender and race; Model 3: adjusted for age, gender, race, education level, the ratio of household income to poverty, BMI, smoking, alcohol, hypercholesterolemia, diabetes, heart failure, coronary heart disease, stroke, ALT, creatinine, uric acid, hemoglobin, iron, SBP and DBP.

ALT = alanine aminotransferase, BMI = body mass index, CI = confidence interval, CRP = C-reactive protein, DBP = diastolic blood pressure, HR = hazard ratio, RAR = red blood cell distribution width-to-albumin ratio, RDW = red blood cell distribution width, Ref = Reference, SBP = systolic blood pressure, WBC= white blood cell.

### 3.3. Stratified and interaction analysis

To investigate whether the results were modified by various comorbidities, subgroup analyses and interactive tests were conducted. The results suggest that no effect modifiers were present (Tables 3 and 4).

**Table 3:** Subgroup analysis and interaction testing (continuous).

	Number	HR (95% CI)	*P* interaction
Hypercholesterolemia			
No	3822	1.61 (1.39, 1.86)	.052
Yes	3008	1.97 (1.60, 2.43)	
Diabetes			
No	5463	1.67 (1.46, 1.90)	.257
Yes	1367	1.76 (1.40, 2.21)	
Heart failure			
No	6407	1.68 (1.48, 1.91)	.500
Yes	423	1.79 (1.26, 2.53)	
Cardiovascular disease			
No	5793	1.72 (1.51, 1.97)	.553
Yes	1037	1.67 (1.31, 2.14)	
Stroke			
No	6345	1.64 (1.43, 1.87)	.313
Yes	485	2.12 (1.59, 2.82)	

Adjusted for age, gender, race, education level, the ratio of household income to poverty, BMI, smoking, alcohol, hypercholesterolemia, diabetes, heart failure, coronary heart disease, stroke, ALT, creatinine, uric acid, hemoglobin, iron, SBP and DBP (excluding stratification factors).

ALT = alanine aminotransferase, BMI = body mass index, CI = confidence interval, DBP = diastolic blood pressure, HR = hazard ratio, SBP = systolic blood pressure .

**Table 4:** Subgroup analysis and interaction testing (quartile).

	Number	HR (95% CI) *P*				*P* trend	*P* interaction
		Q1	Q2	Q3	Q4		
Hypercholesterolemia							
No	3822	Ref.	1.02 (0.79, 1.32)	1.18 (0.91, 1.54)	1.92 (1.48, 2.49)	<.001	.705
Yes	3008	Ref.	1.48 (1.09, 1.99)	1.58 (1.16, 2.16)	2.33 (1.72, 3.15)	<.001	
Diabetes							
No	5463	Ref.	1.16 (0.93, 1.44)	1.23 (0.98, 1.54)	1.94 (1.55, 2.43)	<.001	.806
Yes	1367	Ref.	1.22 (0.80, 1.87)	1.54 (1.00, 2.36)	2.26 (1.49, 3.42)	<.001	
Heart failure							
No	6407	Ref.	1.18 (0.97, 1.44)	1.27 (1.03, 1.57)	1.95 (1.59, 2.40)	<.001	.409
Yes	423	Ref.	1.52 (0.60, 3.84)	2.11 (0.87, 5.13)	3.09 (1.30, 7.36)	<.001	
Cardiovascular disease							
No	5793	Ref.	1.14 (0.92, 1.43)	1.41 (1.12, 1.78)	2.10 (1.67, 2.63)	<.001	.249
Yes	1037	Ref.	1.28 (0.86, 1.92)	1.15 (0.77, 1.72)	1.89 (1.27, 2.81)	.002	
Stroke							
No	6345	Ref.	1.21 (0.99, 1.48)	1.38 (1.12, 1.70)	1.96 (1.59, 2.42)	<.001	.113
Yes	485	Ref.	1.09 (0.58, 2.04)	0.96 (0.50, 1.85)	2.81 (1.54, 5.12)	<.001	

Adjusted for age, gender, race, education level, the ratio of household income to poverty, BMI, smoking, alcohol, hypercholesterolemia, diabetes, heart failure, coronary heart disease, stroke, ALT, creatinine, uric acid, hemoglobin, iron, SBP and DBP (excluding stratification factors).

ALT = alanine aminotransferase, BMI = body mass index, CI = confidence interval, DBP = diastolic blood pressure, HR = hazard ratio, Ref = Reference, SBP = systolic blood pressure .

### 3.4. Sensitivity analysis

In sensitivity analyses, patients were redefined as having hypertension if their blood pressure was ≥130/80 mm Hg or if they were taking antihypertensive medications, based on the 2017 American Heart Association recommendations.^[22]^ A total of 9823 patients with hypertension were ultimately included in the sensitivity analysis. Three extreme values of RAR were excluded to ensure the reliability of the results. The findings did not change significantly compared with those obtained earlier (Table 5).

**Table 5:** Using 130/80 mm Hg to define hypertension (N = 9823).

	Model 1	Model 2	Model 3
	HR (95% CI) *P*	HR (95% CI) *P*	HR (95% CI) *P*
CRP	1.14 (1.11, 1.17) <.001	1.17 (1.14, 1.21) <.001	1.10 (1.06, 1.16) <.001
WBC	1.03 (1.02, 1.04) <.001	1.03 (1.02, 1.04) <.001	1.02 (1.01, 1.04) <.001
Neutrophil	1.12 (1.09, 1.15) <.001	1.18 (1.15, 1.21) <.001	1.12 (1.09, 1.16) <.001
Lymphocyte	0.83 (0.78, 0.88) <.001	1.00 (0.98, 1.03) .860	0.99 (0.95, 1.02) .484
RDW	1.31 (1.29, 1.34) <.001	1.28 (1.25, 1.31) <.001	1.18 (1.14, 1.22) <.001
Albumin	0.36 (0.31, 0.40) <.001	0.40 (0.35, 0.47) <.001	0.54 (0.46, 0.65) <.001
RAR continuous	2.32 (2.18, 2.47) <.001	2.21 (2.05, 2.37) <.001	1.80 (1.61, 2.00) <.001
RAR quartile			
Q1	Ref.	Ref.	Ref.
Q2	1.84 (1.56, 2.17) <.001	1.28 (1.09, 1.51) .003	1.25 (1.04, 1.51) .020
Q3	2.58 (2.21, 3.01) <.001	1.55 (1.33, 1.81) <.001	1.35 (1.13, 1.62) .001
Q4	4.33 (3.74, 5.02) <.001	2.66 (2.28, 3.10) <.001	2.12 (1.75, 2.55) <.001
*P* trend	<.001	<.001	<.001

Model 1: Unadjusted for any covariates; Model 2: adjusted for age, gender and race; Model 3: adjusted for age, gender, race, education level, the ratio of household income to poverty, BMI, smoking, alcohol, hypercholesterolemia, diabetes, heart failure, coronary heart disease, stroke, ALT, creatinine, uric acid, hemoglobin, iron, SBP and DBP.

ALT = alanine aminotransferase, BMI = body mass index, CI = confidence interval, CRP = C-reactive protein, DBP = diastolic blood pressure, HR = hazard ratio, RAR = red blood cell distribution width-to-albumin ratio, RDW = red blood cell distribution width, Ref = Reference, SBP = systolic blood pressure, WBC= white blood cell.

### 3.5. Competitive analysis model

A competing risk model was utilized to explore the impact of RAR on cardiovascular and cerebrovascular disease mortality in patients with hypertension. The results indicated that for every 1 unit increase in RAR, the risk of death from cardiovascular and cerebrovascular diseases increased by 50% (subgroup hazard ratio [SHR] = 1.50, 95% CI: 1.23–1.82; *P* < .001). When RAR was expressed as an interquartile variable, the data suggested that higher RAR levels were associated with an increased risk of death from cardiovascular and cerebrovascular diseases in patients with hypertension (SHR = 1.82; 95% CI: 1.28–2.59; *P* = .001) (Table 6).

**Table 6:** Competing risk model.

	Death of cardiovascular and cerebrovascular diseases	Death of all other cause
	SHR (95% CI) *P*	SHR (95% CI) *P*
RAR continuous	1.50 (1.22, 1.84) <0.001	1.57 (1.31, 1.88) <.001
RAR quartile		
Q1	Ref.	Ref.
Q2	1.27 (0.91, 1.79) .163	1.08 (0.85, 1.36) .540
Q3	1.36 (0.96, 1.92) .086	1.23 (0.96, 1.57) .096
Q4	1.82 (1.28, 2.59) .001	1.83 (1.43, 2.34) <.001
*P* trend	.001	<.001

Adjusted for age, gender, race, education level, the ratio of household income to poverty, BMI, smoking, alcohol, hypercholesterolemia, diabetes, heart failure, coronary heart disease, stroke, ALT, creatinine, uric acid, hemoglobin, iron, SBP and DBP.

ALT = alanine aminotransferase, BMI = body mass index, CI = confidence interval, DBP = diastolic blood pressure, RAR = red blood cell distribution width-to-albumin ratio, Ref = Reference, SBP = systolic blood pressure, SHR = subgroup hazard ratio.

## 4. Discussion

RAR is a novel inflammatory-nutritional biomarker predictive of cardiovascular and cerebrovascular disease prognosis, including acute myocardial infarction, heart failure, and stroke.^[12,14,16]^ Unlike prior hypertension studies limited to isolated RDW or albumin assessments, RAR applicated for hypertension risk stratification in this study. RAR concurrently captures 2 core pathophysiological pathways: inflammatory activation and nutritional imbalance. This dual-pathway quantification provides a more comprehensive reflection of systemic burden than singular biomarkers. Analysis of NHANES data revealed elevated RAR levels correlate with increased 10-year all-cause and cardiovascular disease mortality in patients with hypertensive. Our competing risk models confirmed RAR’s stronger cardiovascular mortality association, while comparative analyses demonstrated its better predictive performance over traditional markers (CRP, WBC, neutrophils, lymphocytes) without additional costs. These findings establish RAR as a cost-effective prognostic tool for hypertension management.

RDW serves as an indicator for assessing the size and variability of circulating red blood cells. Traditionally utilized for diagnosing anemia, recent studies have indicated that RDW can also reflect disease states associated with abnormal erythropoiesis, such as aging, oxidative stress, and systemic inflammation.^[23,24]^ Elevated RDW has been shown to be independently linked to the morbidity and mortality of cardiovascular diseases.^[25–30]^ Previous studies have also reported a relationship between RDW and hypertension.^[17,31–33]^ Inflammation and oxidative stress are believed to be associated with increased RDW levels and an elevated risk of cardiovascular disease.^[34,35]^ The inflammatory response is a significant mechanism contributing to hypertension’s development, promoting endothelial dysfunction, arterial stiffness, vascular remodeling, and target organ damage.^[36]^ Finn Olsen reported on the vascular inflammatory response and inflammatory cell infiltration in hypertensive rats through animal experiments.^[37]^ Under pathological conditions, both innate and adaptive immune cells are activated, releasing cytokines and promoting oxidative stress that leads to kidney and vascular damage, raising blood pressure, and compromising target organs.^[7,38,39]^ Inflammation also serves as a driving factor in the development of atherosclerotic cardiovascular disease.^[40]^ Inflammatory factors contribute to the formation of atherosclerotic plaques and enhance plaque instability through collagen degradation.^[41]^ Wolfram D found that levels of inflammatory factors like tumor necrosis factor-a, interleukin-1, and interleukin-6 were significantly elevated in patients with heart failure.^[42]^ An excessive inflammatory response can hinder bone marrow function and iron metabolism, inhibit erythrocyte production, and result in elevated RDW levels. RDW participates in inflammatory responses, mediating processes that cause vascular endothelial damage, which may explain how RDW influences the prognosis of patients with hypertension. Oxidative stress is another essential mechanism in the development of hypertension, which is characterized by the overproduction of reactive oxygen species and alterations in the redox state. These abnormal conditions can induce protein oxidation and disrupt cell signaling, leading to inflammation, proliferation, apoptosis, migration, and fibrosis, ultimately impairing vascular function and causing cardiovascular remodeling, renal insufficiency, immune cell activation and abnormal excitement of the sympathetic nervous system.^[4,43,44]^ Studies have shown that oxidative stress can directly damage red blood cells and reduce their lifespan, contributing to increased RDW levels.^[13]^ Thus, RDW may hinder blood pressure control through oxidative stress pathways, presenting another potential mechanism by which RDW affects the prognosis of patients with hypertension.

Albumin is a multifunctional protein synthesized by hepatocytes and plays a significant role in various physiological processes in the body. These include maintaining plasma colloid osmotic pressure, transporting various substances, and exhibiting important antioxidant activity. Pathological conditions such as blood volume overload, chronic inflammation, liver congestion, and malnutrition can lead to decreased albumin levels. Low albumin levels are associated with the occurrence of various adverse events, particularly hypertension and cardiovascular disease.^[18,44–50]^ Other studies have shown that low albumin levels correlate with the prognosis of cardiovascular disease.^[44,51,52]^ Albumin levels are associated with nutritional status and inflammatory response,^[44]^ often serving as an indicator of an individual’s nutritional health. Hypoalbuminemia signals poor nutritional status, which can adversely affect patient prognosis. Low albumin is also related to the body’s inflammatory state; studies indicate that albumin levels decrease under inflammatory conditions, with pro-inflammatory mediators such as interleukin-6, interleukin-1, and tumor necrosis factor potentially inhibiting albumin synthesis.^[44,53]^ In patients with chronic inflammation, the liver downregulates albumin mRNA production, leading to reduced synthesis, increased albumin catabolism, and increased vascular permeability, ultimately resulting in lower albumin levels in the body.

RAR, the ratio of RDW to albumin, is a new predictor of inflammation-related diseases. Previous studies have found that RAR is associated not only with the occurrence and prognosis of various diseases.^[12–16,44,52]^ High levels of RAR have been linked to increased mortality in patients with heart failure.^[12]^ A study from Japan demonstrated a significant association between higher RAR and an increased risk of chronic kidney disease (CKD) progression, all-cause mortality, and cardiovascular events in patients with CKD.^[13]^ An other study showed that elevated RAR levels correlate with a heightened risk of 90-day mortality in acute myocardial infarction (AMI) patients.^[14]^ Similarly, higher RAR levels are independently associated with increased all-cause mortality following a stroke.^[16]^ Some studies have indicated that RAR is more effective in predicting the adverse prognosis of aortic aneurysm than RDW and albumin alone.^[15]^ The advantage of RAR is also evident in predicting the prognosis of heart failure.^[12]^ Additionally, other studies have shown that RAR is more accurate than RDW in predicting the risk of contrast-induced nephropathy in patients with ST-elevation myocardial infarction following emergency percutaneous coronary intervention.^[44,54]^

Using the NHANES database, this study found that as RAR levels increase, the 10-year all-cause mortality risk among patients with hypertension also rises. After fully adjusting for confounding factors, these results remained statistically significant (HR: 1.80, 95% CI: 1.61–2.00, *P* < .001), aligning with the findings of several previous studies. In the context of cardiovascular and cerebrovascular diseases, this study discovered through competing risk model analysis that higher RAR levels were associated with a greater risk of death from both cardiovascular and cerebrovascular diseases (SHR = 1.50, 95% CI: 1.22–1.84; *P* < .001). Comparative analysis of established clinical inflammatory markers revealed that RAR demonstrated the strongest association with mortality in Cox regression models. Furthermore, ROC curve analysis confirmed RAR’s better predictive capacity, evidenced by the largest AUC. These findings align with the REGARDS study, which reported CRP limited independent predictive value for hyperntension risk.^[11]^

This study establishes RAR as a novel prognostic indicator for hypertension management. Its implementation advances a dual-pathway framework integrating inflammation control and nutritional optimization. RDW reflects the size heterogeneity of red blood cell, which is influenced by nutritional status, bone marrow function, erythropoietin and other factors. The deficiency of hemoglobin, iron, vitamin B12 and folic acid will affect the level of RDW.^[55]^ For malnutrition patients with low protein level, the intake of high-quality protein should be appropriately increased in the diet, so as to actively improve the nutritional status of patients, which may help improve the prognosis of patients with hypertension.^[56,57]^ Patients with elevated RAR need immediate interventions including intensified blood pressure control, comprehensive nutritional assessment with targeted supplementation, and inflammatory marker profiling. Crucially, RAR derives from routine blood tests without added costs. This enables rapid high-risk population screening and supports its adoption as a practical tool for routine risk stratification and prognostic evaluation in hypertensive cohorts.

However, this study has some limitations. First, the target population was drawn from the NHANES database, which only includes participants from the United States. Thus, the generalizability of these results to other regions requires validation through future studies. Additionally, although this study adjusted for various confounding factors, hypertension is a chronic disease influenced by multiple factors, and it is impossible to completely rule out the effects of imprecisely measured, unknown, or unmeasured confounders on the study results.

## 5. Conclusion

With RAR levels rise, the risk of all-cause mortality and mortality due to cardiovascular disease also escalates. This study suggest that RAR may serve as a useful tool for assessing the prognosis of patients with hypertension.

## Acknowledgments

We would like to thank Editage (www.editage.cn) for English language editing.

## Author contributions

**Data curation:** Jie Li, Yingying Chen.

**Formal analysis:** Suling Ye, Shaomi Li, Jinzhou Zhu, Jie Li.

**Investigation:** Mengmeng Shan, Suling Ye, Shaomi Li.

**Methodology:** Yingying Chen, Jie Li.

**Project administration:** Jie Li.

**Software:** Jie Li.

**Supervision:** Yingying Chen, Jinzhou Zhu, Jie Li.

**Validation:** Mengmeng Shan, Suling Ye, Yingying Chen, Jinzhou Zhu, Jie Li.

**Visualization:** Yingying Chen, Shaomi Li, Jie Li.

**Writing – original draft:** Mengmeng Shan, Suling Ye, Jie Li.

**Writing – review & editing:** Yingying Chen, Shaomi Li, Jinzhou Zhu, Jie Li.
